# Performance of Risk Scores in SARS-CoV-2 Infection: A Retrospective Study

**DOI:** 10.3390/ijerph22081166

**Published:** 2025-07-23

**Authors:** Alessandro Geremia, Arturo Montineri, Alessandra Sorce, Anastasia Xourafa, Enrico Buccheri, Antonino Catalano, Pietro Castellino, Agostino Gaudio, D.O.CoV Research

**Affiliations:** 1 Unit of Infectious Diseases, San Marco Hospital, 95121 Catania, Italy; alegere@hotmail.it (A.G.); a.montineri@ao-ve.it (A.M.); 2 Department of Health Promotion, Mother and Child Care, Unit of Nephrology and Dialysis, Hypertension Excellence Centre, Internal Medicine and Medical Specialties (PROMISE), University of Palermo, 90133 Palermo, Italy; alessandra.sorce@community.unipa.it; 3 Unit of Thalassemia, University Policlinic “G. Rodolico”, 95123 Catania, Italy; axourafa@gmail.com; 4 Unit of Internal Medicine, University Policlinic “G. Rodolico”, 95123 Catania, Italy; pietro.castellino@unict.it (P.C.); agostino.gaudio@unict.it (A.G.); 5 Department of Clinical and Experimental Medicine, University of Messina, 98124 Messina, Italy; catalano.antonino@unime.it; 6 Department of Clinical and Experimental Medicine, University of Catania, 95123 Catania, Italy

**Keywords:** COVID-19, mortality risk, predictive tools, 4C Mortality Score, COVID-GRAM

## Abstract

Prognostic scores that help allocate resources and time to the most critical patients could have potentially improved the response to the SARS-CoV-2 pandemic. We assessed the performance of five risk scores in predicting death or transfer to the intensive care unit (ICU) or sub-intensive care unit (SICU) in hospitalised patients with SARS-CoV-2 infection, with the three aims of retrospectively analysing the effectiveness of these tools, identifying frail patients at risk of death or complications due to infection, and applying these tools in the event of future pandemics. A retrospective observational study was conducted by evaluating data from patients hospitalised with SARS-CoV-2 infection. Among 134 patients considered, 119 were enrolled. All patients were adults, with a mean age of 64 years, and were hospitalised in the Infectious Diseases Division. We compared the five scores using receiver operating characteristic curves and calculation of the areas under the curve (AUCs) to determine their predictive performance. Four of the five scores demonstrated a high accuracy in predicting mortality among COVID-19-positive patients, with AUCs between 0.749 and 0.885. However, only two of the five scores showed good performance in predicting transfer to the ICU or SICU, with AUCs ranging from 0.740 to 0.802. The 4C Mortality Score and COVID-GRAM presented the highest performance for both outcomes. These two scores are easy to apply and low cost. They could still be used in clinical practice as predictive tools for frail and elderly patients with SARS-CoV-2 infection, as well as in the event of future pandemics.

## 1. Introduction

At the beginning of January 2020, a new betacoronavirus, later named SARS-CoV-2, was isolated from bronchoalveolar lavage fluid samples [1]. The virus was first detected in China [2] and spread to other Asian countries before reaching Iran and Italy, where it caused serious epidemics. The World Health Organization (WHO) officially named the disease caused by SARS-CoV-2 as Coronavirus Disease 2019 (COVID-19) [3]. COVID-19 has been a major public health problem, acting as a risk factor for other conditions and contributing, for example, to chronic fatigue syndrome [4] and myopathy [5]. It remains unclear whether low levels of vitamin D play a role in the pathogenic mechanisms of the virus [6]. SARS-CoV-2 placed significant pressure on national healthcare systems, even in industrialised countries, with more than 777 million people infected worldwide and more than 7 million deaths attributed to COVID-19 [7]. However, not all cases were diagnosed, and the actual number of infections and deaths is probably considerably higher. Prognostic scores that enable resources and time to be directed toward the most critical patients could have potentially improved the response to the SARS-CoV-2 pandemic.

For this reason, several prognostic scores have been developed to predict the probability of in-hospital death and the need for escalated treatment. These scores evaluate both laboratory and clinical parameters, stratifying patients into risk classes. The five most widely used and studied scores specifically developed for COVID-19 are the CALL Score [8], the Quick COVID-19 Severity Index (qCSI) [9], the COVID Severi-ty Score [10], the 4C Mortality Score [11], and the COVID-GRAM Critical Illness Risk Score [12].

We retrospectively assessed the performance of these five risk scores in a population of patients hospitalised with SARS-CoV-2 infection. The aims of the study were to retrospectively analyse the effectiveness of these tools, to continue using them for frail patients at risk of death or complications due to infection, and to apply them in future pandemics.

## 2. Materials and Methods

### 2.1. Study Design

The study was conducted through a retrospective evaluation of data from patients with positive SARS-CoV-2 swab results who were admitted to the Division of Infectious Diseases at the University Hospital ‘Policlinico-San Marco’ in Catania between 1 March 2020 and 31 August 2020.

Among 134 adult patients considered, 119 were enrolled, excluding those for whom it was not possible to obtain all the necessary data to calculate the risk scores at the time of admission. The protocol was notified to the local Ethics Committee (Comitato Etico Catania 1, Azienda Ospedaliero-Universitaria Policlinico ‘G. Rodolico—San Marco’ Catania) on 2 December 2020 (protocol number 46180) and was conducted in accordance with the Declaration of Helsinki.

### 2.2. Methods

For each patient included in the study, the following parameters were evaluated at the time of admission: age, sex, presence of chest X-ray abnormalities, presence or absence of haemoptysis or dyspnoea, respiratory rate, peripheral oxygen saturation in ambient air, oxygen therapy administered to maintain adequate SpO_2_ levels (>94%), systolic and diastolic blood pressure, Glasgow Coma Scale score, neutrophil and lymphocyte counts and their ratio, lactate dehydrogenase (LDH), blood urea nitrogen (BUN), direct bilirubin, C-reactive protein (CRP), international normalised ratio, number of comorbidities, and any current or past history of malignancy. Additionally, data were collected on possible outcomes, including death and/or transfer to the intensive care unit (ICU) or sub-intensive care unit (SICU). These parameters were essential for the applicability of the five risk scores. All risk scores were calculated individually for every patient.

### 2.3. Outcome

The primary outcome was defined as all-cause in-hospital mortality, while the secondary outcome was the association between the risk score and the need for transfer to higher-level care units (ICUs and SICUs).

### 2.4. Risk Scores

The CALL score ranges from 4 to 13 points and uses the following variables: comorbidity, age, lymphocyte count, and LDH [8]. The qCSI ranges from 0 to 12, based on the respiratory rate, pulse oximetry, and oxygen flow rate; the values of these three variables must be recorded within the first 4 h of patient admission [9]. The COVID Severity Score uses six parameters: age, oxygen saturation, mean arterial pressure, BUN, CRP, and international normalised ratio. The score ranges from 0 to 10 [10]. The 4C Mortality Score includes eight variables: age, sex, number of comorbidities, respiratory rate, oxygen saturation, Glasgow Coma Scale score, BUN, and CRP. The total score ranges from 0 to 37 [11]. Finally, the COVID-GRAM Critical Illness Risk Score incorporates 10 variables: chest X-ray abnormalities, age, haemoptysis, dyspnoea, unconsciousness, number of comorbidities, cancer history, neutrophil-to-lymphocyte ratio, LDH, and direct bilirubin. The score yields a percentage risk of death or transfer to ICU or SICU, ranging from less than 1.7% to 40.4% or higher [12]. Table 1 visually presents the variables used by each score and the classification into risk categories.

Each score estimates the risk of death or transfer to ICU or SICU for each COVID-19-positive patient, based on the measured parameters, and classifies them into risk categories (see the Results Section), generally from low to high risk, with intermediate levels depending on the specific risk categorisation of each score. These scores were developed using different machine learning techniques, such as gradient boosting decision trees [11] and logistic regression [12]. Machine learning is, in fact, one of the simplest and most accurate methods for predicting disease risk and, in the case of COVID-19, has also made it possible to develop screening tools in a short period of time [11,12]. Indeed, machine learning represents an innovative instrument for the development of screening tools for various diseases, such as diabetes [13,14] or sarcopenia [15,16]. More details on score development and validation are reported in the referenced literature [8,9,10,11,12].

### 2.5. Statistical Analysis

Descriptive statistics were used to summarise patient characteristics, with categorical variables expressed as absolute numbers and percentages and continuous variables as mean ± standard deviation. The diagnostic performance of the five clinical risk scores (CALL, qCSI, COVID Severity Score, 4C Mortality Score, and COVID-GRAM) was evaluated for two outcomes: in-hospital mortality and transfer to the ICU or SICU. Sensitivity, specificity, positive and negative predictive values, positive and negative likelihood ratios, and Youden’s index were calculated for each risk category. Receiver operating characteristic (ROC) curves were generated for each score, and the area under the curve (AUC), with corresponding 95% confidence intervals and standard errors, was calculated to assess discriminatory ability. Model calibration was assessed using the Hosmer–Lemeshow goodness-of-fit test. Based on the Hosmer–Lemeshow classification [17], discrimination was defined as follows: failed for 0.5 ≤ AUC < 0.6, poor for 0.6 ≤ AUC < 0.7, acceptable/good for 0.7 ≤ AUC < 0.8, excellent for 0.8 ≤ AUC < 0.9, and outstanding for AUC ≥ 0.9.

Comparisons between ROC curves were performed using DeLong’s test for two correlated AUCs. Cohen’s kappa (κ) was calculated to assess agreement between the different scoring systems, and its strength of agreement was interpreted according to the Landis and Koch criteria. A *p*-value of <0.05 was considered statistically significant. All statistical analyses were performed using the MedCalc Statistical Software version 23.2.8 (MedCalc Software Ltd., Ostend, Belgium).

## 3. Results

The main characteristics of the enrolled patients are summarised in Table 2. The cohort consisted of 64 men and 55 women, with a mean age of 64.7 ± 18.4 years. A total of 74.8% had at least one comorbidity, the most common being arterial hypertension (47.1%), cardiovascular diseases (19.3%), and neuropsychiatric disorders (16.8%). The in-hospital mortality rate was 19.3%, and 36.9% of the patients were transferred to the highest intensity care unit during hospitalisation.

Table 3 shows the distribution of patients stratified according to the risk categories of each individual score, alongside the actual number of patients who died or were transferred to the ICU or SICU.

Table 4 and Table 5 present the sensitivity, specificity, positive and negative predictive values, and positive and negative likelihood ratios for each risk category of each score, separated by in-hospital mortality and transfers to the ICU or SICU, respectively. Notably, in the low-risk categories for both mortality and transfer to the ICU or SICU, sensitivity was 100% across all scores, but it varied among the different scores in the intermediate and high-risk categories. For example, the CALL score demonstrated 100% sensitivity with 0% specificity in the low-risk mortality category (Class A), whereas in the high-risk mortality category (Class C), sensitivity decreased to 82.6% with a specificity of 66.7%. This pattern suggests that all scores are highly effective at identifying patients truly at low risk of mortality or ICU/SICU transfer (i.e., no false negatives in low-risk categories). By contrast, their ability to correctly identify true positives and true negatives differs in the higher risk categories.

**Figure 1:**
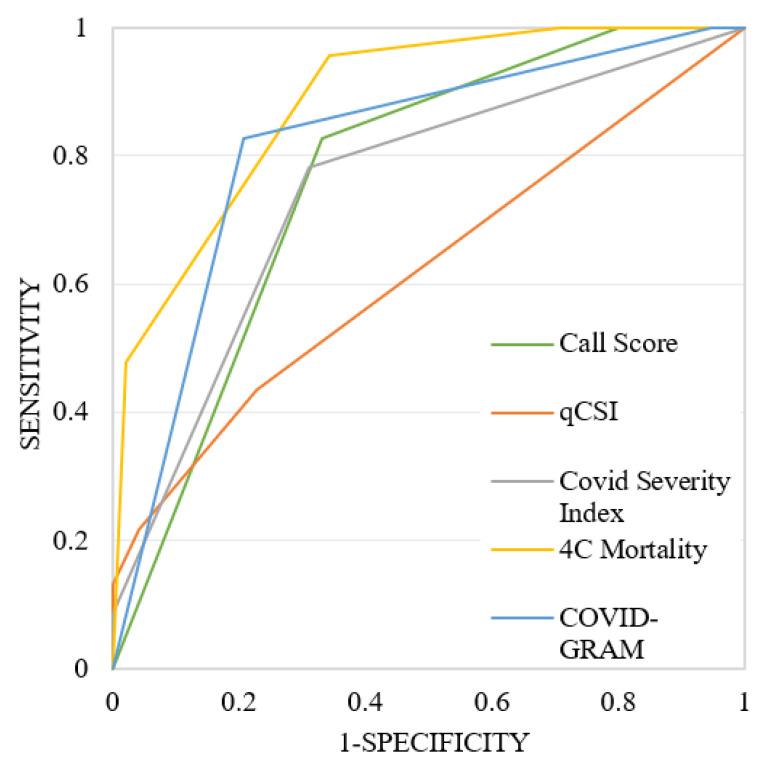
ROC curves of the five analysed scores for predicting in-hospital mortality.

**Figure 2:**
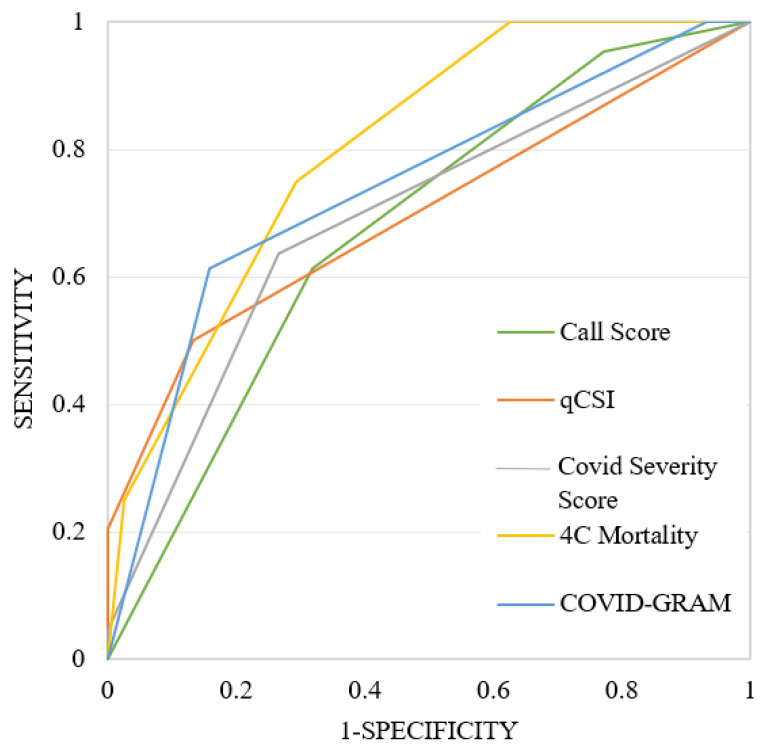
ROC curves of the five analysed scores for predicting transfer to ICU or SICU.

The ROC curves for in-hospital mortality and transfers to the ICU or SICU are shown in Figure 1 and Figure 2, respectively. The results of the AUC calculations, which describe the performance of the scores in our study population, are summarised in Table 6 and Table 7.

The visual analysis of the ROC curves (Figure 1 and Figure 2) and the AUC values (Table 6 and Table 7) showed that the 4C Mortality Score had greater accuracy in predicting both in-hospital mortality and transfers to ICU or SICU than the other four scores, with the COVID-GRAM being the only score close in accuracy for predicting in-hospital mortality and slightly less accurate for predicting transfers to ICU or SICU.

Subsequently, the ROC curves were compared pairwise to assess whether there was statistically significant superiority of one score over another, as well as to evaluate the degree of agreement between scores using Cohen’s kappa, for both in-hospital mortality risk (Table 8) and transfer to the ICU or SICU risk (Table 9).

The comparison of AUCs showed similar accuracy among the CALL, qCSI, COVID Severity Score, and COVID-GRAM, while the 4C Mortality Score demonstrated significantly superior accuracy compared with the first three scores for mortality risk and compared with the CALL and COVID Severity Score for predicting the risk of transfer to the ICU or SICU.

## 4. Discussion

In this study, we evaluated the performance of the CALL Score, qCSI, COVID Severity Score, 4C Mortality Score, and COVID-GRAM in predicting in-hospital mortality and the need for higher-intensity treatment in our cohort of patients hospitalised for COVID-19. With regard to mortality prediction, the 4C Mortality Score clearly demonstrated the best performance (AUC, 0.885; *p* < 0.001), showing excellent reliability similar to that of COVID-GRAM (AUC, 0.813; *p* < 0.001).

The same applies to reliability in predicting the need for intensive care: the 4C Mortality Score slightly exceeded the cut-off for excellent reliability (AUC, 0.802; *p* < 0.001), while COVID-GRAM still showed good reliability (AUC, 0.740; *p* < 0.001). Both scores demonstrated comparable [18,19,20,21] or even better performance in our population than reported in previous studies [22,23,24]. For example, a study conducted in Ontario, Canada, on 959 patients hospitalised between March 2020 and June 2021 found the 4C Mortality Score to be highly reliable during the first wave, with a slight decrease in discriminatory power during the second and third waves (AUC < 0.800), yet still maintaining good accuracy [25]. Furthermore, the 4C Mortality Score has been shown to maintain excellent performance even in the presence of COVID-19 variants such as Omicron [20]. Notably, preliminary data published in a letter to the editor in the *Journal of Infection* (October 2020) [26] by Z. Wellbelove and colleagues compared the performance of the 4C Mortality Score to other well-known and validated scores not specific to COVID-19—such as CURB65, CRB65, NEWS, and qSOFA—applied not only to COVID-19 but also to influenza, community-acquired pneumonia, and invasive pneumococcal infection. The 4C Mortality Score showed good reliability across all contexts, whereas the other scores performed better specifically in patients with influenza. Regarding COVID-GRAM, our results show higher accuracy than in a Spanish cohort of hospitalised patients between March and May 2020 (AUC, 0.720) and align with the Chinese validation cohort of the score (AUC, 0.880) [24].

The COVID Severity Score and the CALL Score demonstrated good performance, consistent with findings reported in the literature [10,27,28]. In particular, the results obtained using the COVID Severity Score overlap with those of the validation study [10]. In a letter to the editor published in *Clinical Infectious Diseases* (January 2021), E. Grifoni and colleagues reported that the CALL Score showed good reliability in predicting in-hospital mortality (AUC, 0.768), but only sufficient accuracy in predicting progression to severe disease (AUC, 0.622) [27]—a trend also observed in our study. Conversely, in a cohort of unvaccinated Chilean patients with COVID-19, the CALL Score showed high accuracy in predicting 12-month mortality (AUC, 0.862) [28]. In our population, the score with the lowest predictive accuracy was the qCSI, which performed worse than reported in previous studies [9,29,30,31]. In fact, all previous studies have shown good performance for the qCSI, with AUC values consistently higher than 0.700 for predicting both mortality risk and the need for intensive care. This discrepancy is probably due to the fact that the qCSI was originally designed to identify the risk of death and the need for intensive care within 24 h of score measurement. Therefore, calculating the score only at the time of hospital admission—as in our study, conducted in an infectious diseases ward—inevitably reduces its predictive reliability. By contrast, in emergency department or intensive care settings, this score may perform better because it is applied immediately to assess the risk of death or the need for higher-intensity care. Nonetheless, in our study, the qCSI showed a better tendency in predicting the risk of transfer to ICU or SICU, reaching almost the cut-off for good reliability (AUC, 0.697; *p* < 0.001), compared with its lower performance in predicting mortality (AUC, 0.621; *p* = 0.046). We chose to include the qCSI in our study, despite its limitations, given its applicability in non-ICU settings and because it remains one of the most frequently reported scores in the literature.

Based on the comparison of the ROC curves, the 4C Mortality Score showed statistically significant superiority over the CALL Score and COVID Severity Score for both outcomes and over the qCSI for the primary outcome only. Additionally, COVID-GRAM demonstrated statistically significant superiority over qCSI for the primary outcome. According to the Cohen’s kappa analysis, a moderate agreement was observed between the CALL Score and 4C Mortality Score (Cohen’s kappa = 0.50), as defined by the Landis and Koch scale.

Finally, all the evaluated scores proved useful in identifying COVID-19-positive patients at a higher risk of disease progression and in-hospital mortality. However, the 4C Mortality Score and COVID-GRAM demonstrated superior and consistently excellent performance. These two scores, together with established clinical monitoring parameters, could serve as valuable tools for predicting the risk of clinical deterioration or death in patients with COVID-19, both in internal medicine wards and in ICUs.

The limitations of our study include the relatively small sample size, the retrospective design, and the single-centre setting. It is important to underline the need for larger, prospective, multicentre studies to assess the exact reliability of these scores and to evaluate their applicability to other infectious respiratory diseases. Other limitations include the possible influence of patients’ vaccination status, as well as infection with specific COVID-19 variants (Delta, Omicron, etc.). These factors should be explored in dedicated studies.

## 5. Conclusions

The 4C Mortality Score and COVID-GRAM demonstrated excellent performance, significantly outperforming the other scores by approximately 10% in AUC for predicting in-hospital mortality or transfer to the ICU or SICU in COVID-19-positive patients. These easy-to-apply, low-cost scores could still be used in clinical practice as predictive tools in frail and elderly patients, who are therefore at higher risk of complications or death due to SARS-CoV-2 infection. While our findings support the use of the 4C Mortality Score and COVID-GRAM, further validation is needed given the limitations of our study. Multicentre studies with larger patient cohorts are recommended to more accurately assess the performance of the five evaluated scores. In addition, stratified analyses should be conducted to evaluate the effectiveness of these scores in specific settings, such as according to patients’ vaccination status or the SARS-CoV-2 variant involved.

A future objective is to apply these scores not only in the management of seasonal epidemics but also in the event of future pandemics.

## Figures and Tables

**Table 1:** Risk scores.

Type of Score	Risk Class Classification	Variables Used
CALL score [8]	Class A (4–6) Class B (7–9) Class C (10–13)	Comorbidity Age Lymphocyte LDH
qCSI [9]	Low (0–3) Low-medium (4–6) Medium-high (7–9) High (10–12)	Respiratory rate Pulse oximetry Oxygen flow rate
COVID Severity Score [10]	Low (0–3) Medium (4–7) High (8–10)	Age Oxygen saturation Mean arterial pressure BUN CRP INR
4C Mortality Score [11]	Low (0–3) Medium (4–8) High (9–14) Very high (≥15)	Age Gender Number of comorbidities Respiratory rate Oxygen saturation GCS BUN CRP
COVID-GRAM [12]	Low (<1.7%) Medium (1.7% to <40.4%) High (≥40.4%)	Chest X-ray abnormalities Age Haemoptysis Dyspnoea Unconsciousness Number of comorbidities Cancer history NLR LDH Direct bilirubin

**Table 2:** Patient characteristics. Data are expressed as number (%) or mean ± standard deviation.

Male	64 (53.8)
Female	55 (46.2)
Age (years)	64.7 ± 18.4
Any Comorbidity	89 (74.8)
Hypertension	56 (47.1)
Diabetes	15 (12.6)
CKD	13 (10.9)
Cardiovascular diseases	23 (19.3)
Neuropsychiatric diseases	20 (16.8)
COPD	6 (5)
Cancer	7 (5.9)
Chest X-ray abnormalities	107 (89.9)
Dyspnoea	54 (45.4)
In-hospital Mortality	23 (19.3)
Transferred to ICU/SICU	44 (36.9)

**Table 3:** In-hospital mortality and transfers to the ICU or SICU according to the risk categories of the scores.

CALL Score	No. of Patients	Deaths	Transferred
Class A (4–6)	19	0	2
Class B (7–9)	49	4	15
Class C (10–13)	51	19	27
qCSI	No. of patients	Deaths	Transferred
Low (0–3)	87	13	22
Low–medium (4–6)	23	5	13
Medium–high (7–9)	6	2	6
High (10–12)	3	3	3
COVID Severity Score	No. of patients	Deaths	Transferred
Low (0–3)	71	5	16
Medium (4–7)	46	16	26
High (8–10)	2	2	2
4C Mortality Score	No. of patients	Deaths	Transferred
Low (0–3)	28	0	0
Medium (4–8)	36	1	11
High (9–14)	42	11	22
Very high (≥ 15)	13	11	11
COVID-GRAM	No. of patients	Deaths	Transferred
Low (<1.7%)	5	0	0
Medium (1.7% to <40.4%)	75	4	17
High (≥40.4%)	39	19	27

**Table 4:** Sensitivity, specificity, positive predictive value, negative predictive value, likelihood ratios, and Youden’s index for in-hospital mortality.

Score	Risk Score	Sensitivity (95% CI)	Specificity (95% CI)	PPV (95% CI)	NPV (95% CI)	(+)LR (95% CI)	(-)LR (95% CI)
CALL Score	Class A	100 (85.2–100)	0.0 (0.0–3.8)	19.3	-	1.00	-
	Class B	100 (85.2–100)	19.8 (12.4–29.2)	23 (21.3–24.8)	100	1.25 (1.13–1.38)	0.00
	Class C	82.6 (61.2–95)	66.7 (56.3–76)	37.3 (29.7–45.5)	94.1 (86.7–97.5)	2.48 (1.76–3.48)	0.26 (0.11–0.64)
qCSI	Low	100 (85.2–100)	0.0 (0.0–3.8)	19.3	-	1.00	-
	Low–medium	43.5 (23.2–65.5)	77.1 (67.4–85.0)	31.3 (20.1–45.1)	85.1 (79.6–89.2)	1.90 (1.05–3.43)	0.73 (0.50–1.07)
	Medium–high	21.7 (7.5–43.7)	95.8 (89.7–98.9)	55.6 (26.7–81.1)	83.6 (80.4–86.4)	5.22 (1.52–17.91)	0.82 (0.66–1.02)
	High	13.0 (2.8–33.6)	100 (96.2–100)	100	82.8 (80.4–84.9)	-	0.87 (0.74–1.02)
COVID Severity Score	Low	100 (85.2–100)	0.0 (0.0–3.8)	19.3	-	1.00	-
	Medium	78.3 (56.3–92.5)	68.8 (58.5–77.8)	37.5 (29.4–46.4)	93.0 (85.7–96.7)	2.50 (1.74–3.61)	0.32 (0.14–0.69)
	High	8.7 (1.1–28.0)	100 (96.2–100)	100	82.1 (80.1–83.8)	-	0.91 (0.80–1.04)
4C Mortality Score	Low	100 (85.2–100)	0.0 (0.0–3.8)	19.3	-	1.00	-
	Medium	100 (85.2–100)	29.2 (20.3–39.3)	25.3 (22.9–27.8)	100	1.41 (1.24–1.61)	0.00
	High	95.7 (78.1–99.9)	65.6 (55.2–75.0)	40.0 (33.3–47.1)	98.4 (90.2–99.8)	2.78 (2.08–3.72)	0.07 (0.01–0.45)
	Very high	47.8 (26.8–69.4)	97.9 (92.7–99.7)	84.6 (56.7–95.9)	88.7 (84.1–92.1)	22.96 (5.46–96.54)	0.53 (0.36–0.79)
COVID-GRAM	Low	100 (85.2–100)	0.0 (0.0–3.8)	19.3	-	1.00	-
	Medium	100 (85.2–100)	5.2 (1.7–11.7)	20.2 (19.4–20.9)	100	1.05 (1.01–1.11)	0.00
	High	82.6 (61.2–95.0)	79.2 (69.7–86.8)	48.7 (38.1–59.4)	95.0 (88.6–97.9)	3.97 (2.57–6.11)	0.22 (0.09–0.54)

**Table 5:** Sensitivity, specificity, positive predictive value, negative predictive value, and likelihood ratios for transfers to ICU or SICU.

Score	Risk Score	Sensitivity (95% CI)	Specificity (95% CI)	PPV (95% CI)	NPV (95% CI)	(+)LR (95% CI)	(-)LR (95% CI)
CALL Score	Class A	100 (92.0–100)	0.0 (0.0–4.8)	37.0	-	1.00	-
	Class B	95.5 (84.5–99.4)	22.7 (13.8–33.8)	42.0 (38.7–45.4)	89.5 (67.3–97.2)	1.23 (1.07–1.42)	0.20 (0.05–0.83)
	Class C	61.4 (45.5–75.6)	68.0 (56.2–78.3)	52.9 (42.9–62.8)	75.0 (66.7–81.8)	1.92 (1.28–2.87)	0.57 (0.38–0.85)
qCSI	Low	100 (92.0–100)	0 (0–4.8)	37.0	-	1.00	-
	Low–medium	50.0 (34.6–65.4)	86.7 (76.8–93.4)	68.8 (53.5–80.8)	74.7 (68.5–80.1)	3.75 (1.96–7.17)	0.58 (0.42–0.79)
	Medium–high	20.5 (9.8–35.3)	100 (95.2–100)	100	68.2 (64.8–71.3)	-	0.80 (0.68–0.92)
	High	6.8 (1.4–18.7)	100 (95.2–100)	100	64.7 (62.8–66.5)	-	0.93 (0.86–1.01)
COVID Severity Score	Low	100 (92.0–100)	0.0 (0.0–4.8)	37.0	-	1.00	-
	Medium	63.6 (47.8–77.6)	73.3 (61.9–82.9)	58.3 (47.5–68.4)	77.5 (69.4–83.9)	2.39 (1.54–3.69)	0.50 (0.33–0.75)
	High	4.6 (0.6–15.5)	100 (95.2–100)	100	64.1 (62.6–65.6)	-	0.95 (0.89–1.02)
4C Mortality Score	Low	100 (92.0–100)	0.0 (0.0–4.8)	37.0	-	1.00	-
	Medium	100 (92.0–100)	37.3 (26.4–49.3)	48.4 (44.0–52.7)	100	1.60 (1.34–1.90)	0.00
	High	75.0 (59.7–86.8)	70.7 (59.0–80.6)	60.0 (50.4–68.9)	82.8 (73.9–89.1)	2.56 (1.73–3.78)	0.35 (0.21–0.60)
	Very high	25.0 (13.2–40.3)	97.3 (90.7–99.7)	84.6 (56.1–95.9)	68.9 (65.0–72.5)	9.38 (2.18–40.37)	0.77 (0.65–0.92)
COVID-GRAM	Low	100 (92.0–100)	0.0 (0.0–4.8)	37.0	-	1.00	-
	Medium	100 (92.0–100)	6.7 (2.2–14.9)	38.6 (37.2–40.0)	100	1.07 (1.01–1.14)	0.00
	High	61.4 (45.5–75.6)	84.0 (73.7–91.4)	69.2 (56.0–79.9)	78.7 (78.6–84.5)	3.84 (2.17–6.78)	0.46 (0.31–0.68)

**Table 6:** AUC of ROC curves, standard error, and confidence interval for the five scores for in-hospital mortality, with corresponding statistical significance.

In-Hospital Mortality	AUC	SE	95% CI	*p* Value
CALL Score	0.764	0.041	0.677 to 0.837	<0.001
qCSI	0.621	0.061	0.528 to 0.709	0.046
COVID Severity Score	0.749	0.051	0.661 to 0.824	<0.001
4C Mortality	0.885	0.031	0.814 to 0.936	<0.001
COVID-GRAM	0.813	0.044	0.732 to 0.879	<0.001

**Table 7:** AUC of ROC curves, standard error, and confidence interval for the five scores for transfers to ICU or SICU, with corresponding statistical significance.

ICU Admission	AUC	SE	95% CI	*p* Value
CALL Score	0.675	0.045	0.583 to 0.758	<0.001
qCSI	0.697	0.043	0.606 to 0.778	<0.001
COVID Severity Score	0.691	0.045	0.600 to 0.772	<0.001
4C Mortality	0.802	0.037	0.719 to 0.869	<0.001
COVID-GRAM	0.740	0.041	0.651 to 0.816	<0.001

**Table 8:** Pairwise comparison of AUCs for each score, including AUC differences, standard error, confidence interval, statistical significance, and inter-rater reliability (Cohen’s kappa) for in-hospital mortality risk.

Score Comparison	|AUC1-AUC2|	SE	95% CI	*p* Value	Cohen’s K
CALL Score~qCSI	0.142	0.075	−0.004 to 0.289	0.057	0.034
CALL Score~CSS	0.015	0.052	−0.086 to 0.116	0.772	0.148
CALL Score~4C Mortality	0.121	0.042	0.038 to 0.204	**0.004**	0.509
CALL Score~COVID-GRAM	0.050	0.061	−0.069 to 0.169	0.413	0.367
qCSI~CSS	0.127	0.075	−0.020 to 0.274	0.089	0.205
qCSI~4C Mortality	0.264	0.070	0.127 to 0.400	**<0.001**	0.125
qCSI~COVID-GRAM	0.192	0.076	0.049 to 0.341	**0.012**	0.052
CSS~4C Mortality	0.136	0.040	0.058 to 0.214	**<0.001**	0.185
CSS~COVID-GRAM	0.065	0.069	−0.071 to 0.201	0.350	0.061
4C Mortality~COVID-GRAM	0.072	0.052	−0.030 to 0.173	0.166	0.350

Bold indicates significant *p* values.

**Table 9:** Pairwise comparison of AUCs for each score, including AUC differences, standard error, confidence interval, and statistical significance for transfer to ICU or SICU risk.

Score Comparison	|AUC1-AUC2|	SE	95% CI	*p* Value
CALL Score~qCSI	0.022	0.064	−0.104 to 0.148	0.734
CALL Score~CSS	0.016	0.041	−0.064 to 0.096	0.699
CALL Score~4C Mortality	0.127	0.037	0.054 to 0.199	**<0.001**
CALL Score~COVID-GRAM	0.065	0.050	−0.033 to 0.162	0.193
qCSI~CSS	0.006	0.059	−0.109 to 0.121	0.918
qCSI~4C Mortality	0.105	0.056	−0.005 to 0.215	0.063
qCSI~COVID-GRAM	0.043	0.064	−0.083 to 0.168	0.505
CSS~4C Mortality	0.111	0.035	0.042 to 0.180	**0.002**
CSS~COVID-GRAM	0.049	0.049	−0.048 to 0.146	0.324
4C Mortality~COVID-GRAM	0.062	0.043	−0.022 to 0.146	0.146

Bold indicates significant *p* values.

## Data Availability

The data presented in this study are available upon request from the corresponding author.

## Abbreviations

(AUC)	Area under the curve
(BUN)	Blood urea nitrogen
(CKD)	Chronic kidney disease
(COPD)	Chronic obstructive pulmonary disease
(CAP)	Community-acquired pneumonia
(COVID-19)	Coronavirus disease 2019
(CRP)	C-reactive protein
(GCS)	Glasgow Coma Scale
(ICU)	Intensive care unit
(INR)	International normalised ratio
(LDH)	Lactate dehydrogenase
(-)(LR)	Negative likelihood ratio
(NPV)	Negative predictive value
(NLR)	Neutrophil and lymphocyte values and their ratio
(+)(LR)	Positive likelihood ratio
(PPV)	Positive predictive value
(ROC)	Receiver operating characteristic
(SICU)	Sub-intensive care unit
